# Teenage pregnancy rates and associations with other health risk behaviours: a three-wave cross-sectional study among South African school-going adolescents

**DOI:** 10.1186/s12978-016-0170-8

**Published:** 2016-05-04

**Authors:** Kim Jonas, Rik Crutzen, Bart van den Borne, Ronel Sewpaul, Priscilla Reddy

**Affiliations:** School of Public Health and Primary Care (CAPHRI), Health Promotion Department, Maastricht University, P.O. Box 616, Maastricht, 6200 MD The Netherlands; Human Sciences Research Council (HSRC), Population Health, Health Systems and Innovation Unit, Cape Town, South Africa; Faculty of Community and Health Science, Social Work Department, Child and Family Studies, University of the Western Cape, Cape Town, South Africa

**Keywords:** Teenage pregnancy, Risky sexual behaviours, Pregnancy, Adolescents, Health risk behaviours, Trends, Adolescent reproductive health, Substance use

## Abstract

**Background:**

Teenage pregnancy still remains high in low and middle-income countries (LMIC), as well as in high-income countries (HIC). It is a major contributor to maternal and child morbidity and mortality rates. Furthermore, it has social consequences, such as perpetuating the cycle of poverty including early school dropout by the pregnant adolescent, especially in sub-Saharan Africa (SSA). Few studies in SSA have investigated the trends in teenage pregnancy and the associated factors, while this is critical in fully understanding teenage pregnancy and for promotion of reproductive health among adolescents at large in SSA.

**Methods:**

To examine the trends in teenage pregnancy and to identify associations with other health risk behaviours in South Africa (SA), a total of 31 816 South African school-going adolescents between 11 to 19 years of age were interviewed in three cross-sectional surveys. Data from the first (2002, *n* = 10 549), second (2008, *n* = 10 270) and the third (2011, *n* = 10 997) nationally representative South African youth risk behaviour surveys (YRBS) were used for this study.

**Results:**

The overall prevalence of having ever been pregnant among the combined 3-survey sample was self-reported to be 11.0 % and stable across the three surveys. Sexual intercourse among adolescents in SA has decreased from 41.9 % in 2002 to 36.9 % in 2011. However, pregnancy among girls who ever had sex increased from 17.3 % (95 % CI: 0.16–0.19) in 2002, to 23.6 % (95 % CI: 0.21–0.26) in 2008 and decreased to 21.3 % (95 % CI: 0.19–0.23) in 2011. The odds for ever been pregnant were higher for girls who had 2 or more sexual partners (OR: 1.250, 95 % CI: 1.039–1.503), girls who ever used alcohol before sex (OR: 1.373, 95 % CI: 1.004–1.878), practised binge-drinking during the last month (OR: 0.624, 95 % CI: 0.503–0.774), and girls who used mandrax (OR: 1.968, 95 % CI: 1,243–3.117). The odds for never been pregnant were lower for those who used condoms (OR: 0.462, 95 % CI: 0.309–0.691).

**Conclusions:**

Girls continue to become pregnant at unacceptably high rates in SA. Sexual intercourse among adolescents in SA has decreased slightly. However, among those who are sexually active pregnancy prevalence rates have increased. More over, this is in the context of high prevalence of HIV and other STI. There is a need to address adolescents’ sexual and reproductive health, and several health risk behaviours, including substance use, that are associated with teenage pregnancy in SA.

## Background

High teenage pregnancy rates are reported in both LMICs and HICs. The rate of teenage pregnancy in the United States is among the highest (at about 24 %) of the HICs [1] while more than 50 % of all births occurring during adolescence are in sub-Saharan Africa [2]. Globally, almost 16 million girls aged 15 to 19 give birth every year [2] and about 2.5 million of these births occur to girls aged under 16 in LMICs each year [3]. This is about 11 % of all births worldwide, with the majority of these (95 %) occurring in LMIC [2]. Complications during pregnancy and childbirth worldwide are the second leading cause of death for 15–19 year-old girls, with girls below 16 years of age at a higher risk for maternal mortality and severe morbidity in comparison with women above the age of 20 years [2–6]. In addition, birth outcomes for very young adolescent mothers are particularly poor in terms of increased rates of low birth weights [6, 7]. Furthermore, teenage pregnancy is a major contributor to a never ending cycle of ill-health and poverty worldwide [2, 4–6].

Teenage pregnancy in SA is a multifaceted problem with many contributing factors such as poverty, gender inequalities, gender-based violence, substance use, poor access to contraceptives and issues with termination of pregnancy; low, inconsistent and incorrect use of contraceptives, limited number of healthcare practitioners and healthcare facilities, poor healthcare workers’ attitudes and behavior, and inadequate sexual and reproductive health (SRH) information [6–9]. Besides these structural and healthcare systems related factors, adolescents in general tend to engage in risky behaviours including sexual activities that eventually put them at risk for unwanted pregnancies, sexually transmitted infections (STI’s), and HIV [10]. Sexual risk-taking behaviours, including early sexual debut, unprotected sex, multiple sex-partners and low contraceptive use are common among young people in SA [10–12], even though the SA government has made the provision of contraceptives free since 2001. The government continues to update and upgrade the scope of contraceptives in the country, including the addition of the very modern forms of contraception such as implants that are available in the public state hospitals and in primary healthcare clinics since 2014 [13–15]. Since 1994, emergency contraceptives and maternal and child healthcare services are free of charge while termination of pregnancy (TOP) has been legal and is also provided free of charge since 1996.

There exists a large body of literature worldwide on factors associated with teenage pregnancy and early childbearing both in HIC and in LMIC, such as low levels of contraceptive use. For example, a study on contraceptive use among adolescents in Kenya reported the use to be low, despite the reported high levels of knowledge about contraceptives among this population [16]. In SA, a few studies have also reported on the factors associated with teenage pregnancy and other sexual risk behaviours such the low use of contraceptives and poor knowledge of contraceptives, amongst others mentioned above [12, 17–20]. The reproductive health ignorance among young people, such as the basic understanding of a pregnancy possibility as a consequence of unsafe sexual intercourse, is also contributing to teenage pregnancy [21]. Unsafe sex is common among South African adolescents and is known to result in a number of unpleasant health and social consequences [9, 12, 20–24]. Teenage pregnancy in SA is on top of the list of consequences of unsafe sex, following HIV and AIDS, and other STI’s.

Sexual risk behaviors and substance use often occur in combination with one another, both in developed countries as well as in LMIC [25–28]. Substance use, particularly marijuana and cocaine, continue to represent individual-level risk factors associated with adolescents sexual behaviors, including use of contraception, safe sex practices, and teenage pregnancy [25]. Furthermore, substance use among adolescents, especially alcohol and drugs has been reported to influence what adolescents do sexually, and makes them less likely to practice safe sex [26]. Substance use behaviors in general, independently contribute to an increased risk in sexual intercourse experience with and without a history of teenage pregnancy [25, 26]. A dose-response relationship between an increased likelihood of a teenage pregnancy and both marijuana use and daily cigarette smokers who initiated use at age 12 or younger was reported among adolescents in the United States (US) [26]. Substance use in SA has been associated with higher odds of lifetime sexual intercourse [27]. High school adolescents who used alcohol or smoked cigarettes were two to three times more likely to be sexually active in the KwaZulu-Natal province [28].

There is clearly a lot of literature on teenage pregnancy, but its scope is limited to the causes of teenage pregnancy [21], the implications of pregnancy to the young mother (and her baby) [22, 29], and to school dropout related factors [22, 24]. There are however, very limited studies published that report on recent trends in teenage pregnancy in SA [29]. Even less is known about the factors associated with trends in teenage pregnancy. Analysis of teenage pregnancy trends and the associated factors is of paramount importance, as it will help to understand fully the factors surrounding teenage pregnancy in SA. It provides useful information towards understanding the magnitude of teenage pregnancy among the South African youth and helps determining areas that require serious intervention efforts in order to reduce the rates of teenage pregnancy. Understanding the factors or determinants associated with teenage pregnancy is crucial because it is this understanding that will enable development of intervention programmes needed to address teenage pregnancy in the country. Thus, the aim of this particular study is to examine the trends in teenage pregnancy as well as related health risk behaviors, including unsafe sex, substance use, partner violence, and psychological well-being; which might act as contributory factors associated with teenage pregnancy among school-going adolescents between 11 and 19 years of age in different settings of the country.

## Methods

Data of the three *Umthente Uhlaba Usamila*: South African National Youth Risk Behaviour Surveys (YRBS) are used in this study. Three cross-sectional surveys were conducted among nationally representative samples of grades 8 to 11 secondary school adolescents in South Africa in 2002, 2008, and 2011.

## Participants and sampling

The SA YRBS used a two-stage cluster sample design. At the first stage of sampling, schools were stratified according to the country’s nine provinces. For each sample the most recent list of all public schools in the country, provided by the South African National Department of Education, was used as the sampling frame, to ensure nationally representative data. The schools were the primary sampling units and were selected with a probability proportional to the schools’ adolescent enrolment in grades 8 to 11. In each survey twenty-three schools per province were sampled and invited to participate. At the second stage of sampling, classes were randomly selected within each participating school. A total of 14 766 (for 2002 survey), 13 379 (for the 2008 survey) and 14 387 (for the 2011 survey) enrolled adolescents from grades 8–11 in the participating schools per province were invited for each survey respectively. Nationally, a 72 % response rate for 2002 and 2011, and 76 % for 2008 was achieved at the adolescent level.

Adolescents completed a self-administered questionnaire on a range of socio-demographic characteristics and risk behaviour variables. The YRBS questionnaire was adapted from the US Youth Risk Behaviour Survey, a health survey conducted biennially among high school adolescents in the United States [30]. The measures used in the study are specified below. Further details regarding the South African YRBS survey and methodology can be found elsewhere [31–34].

## Measures

### Demographics

*Age:* to determine the ages of adolescents in the sample, adolescents were asked to report both their age and date of birth.

*Gender:* adolescents were asked to report their gender (0 = Male, 1 = Female).

*Race:* race was classified according to the South African Department of Labour designated categories: Black African (=1), Coloured (mixed Black and White descent (=2), Indian (=3), White (=4), or Other (=5).

### Socio-economic status

*Fathers’ employment status*: if adolescents reported their father having a paying job 5 or more days a week (=1), No, he gets a social grant (=2), No, he is unemployed (=3), No, he is ill or disabled (=4), No, he has retired (=5), My father is deceased (=6), I don’t know (=7).

*Mothers’ employment status:* if adolescents reported their mother having a paying job 5 or more days a week (=1), No, she is a housewife (=2), No, she is unemployed (=3), No, she is ill or disabled (=4), No, she has retired (=5), My mother is deceased (=6), I don’t know (=7).

### Sexual behaviours

*Ever had sex*: if adolescents reported having ever had sex (when the penis enters the vagina or anus) (1 = Yes, 0 = No). The following questions were dependent on whether the adolescent answered “yes” (1) on ever had sex, although there was no skip pattern for adolescents who answered “no” (0).

*First had sex before age of 14 years*: if adolescents reported having had first sex at the age of 13 years or younger (1 = had sex at ≤ 13 years, 0 = have never had sex or had sex at age 14 years or older).

*Number of lifetime sexual partners*: if adolescents reported having ever had sex with 1 or more people (0 = never had sex or 1 partner, 1 = 2 or more partners). For the purposes of this study, lifetime sexual intercourse is defined as the number of times whereby one’s penis had penetrated a vagina (or one’s vagina ever been penetrated by a penis) since they were born. The number of lifetime sexual partners therefore, refers to the number of sexual partners one has ever had sexual intercourse with in their lifetime.

*Used alcohol before sex:* if adolescents reported having consumed alcohol before the last time they had sex (1 = Yes, 0 = No/never had sex).

*Always or mostly used a condom during sex:* if adolescents reported always using condoms when they have sex (1 = always or mostly using a condom before sex, 0 = never had sex or not always using a condom during sex or never using a condom during sex).

*Contraceptive methods mostly used to prevent pregnancy*: if adolescents reported using contraceptives (0 = No method, 1 = Birth control pills, 2 = Condoms, 3 = Contraceptive injection, 4 = Other).

*Ever been pregnant*: if adolescents reported to ever been pregnant (1 = Yes, 0 = No/never had sex).

*Ever had an abortion*: if adolescents reported to ever had an abortion (1 = Yes, 0 = No/never sex/never pregnant).

### Violent behaviours

*Ever been hit by boyfriend or girlfriend*: if adolescents reported that, during the past 6 months, their boyfriend or girlfriend hit, smacked or physically hurt them on purpose, then they were classified as being physically hurt by boyfriend or girlfriend (1 = Yes, 0 = No).

*Ever hit your boyfriend or girlfriend*: if adolescents reported that, during the past 6 months, they have hurt their boyfriend or girlfriend, smacked or physically hurt them on purpose, then they were classified as having physically hurt their boyfriend or girlfriend (1 = Yes, 0 = No).

*Ever been forced to have sex*: if adolescents reported that they had ever been physically forced to have sex when they did not want to, then they were classified as such (1 = Yes, 0 = No).

*Ever forced someone to have sex*: if adolescents reported that they had ever physically forced someone to have sex when they did not want to, then they were classified as such (1 = Yes, 0 = No).

### Substance use behaviours

*Current cigarette smoking:* Adolescents were categorised as being current cigarette smokers if they reported having smoked cigarettes on at least one day in the past month (1 = Yes, 0 = No).

*Alcohol use:* Adolescents were categorised as being alcohol users if they reported having ever drank one or more drinks of alcohol (1 = Yes, 0 = No) in their lifetime.

*Binge drinking in the past month:* Adolescents were categorised as having engaged in binge drinking if they reported having had five or more drinks of alcohol (e.g. a beer, a glass of wine, or a ‘tot’ of brandy) in a row on at least one day in the past month (1 = Yes, 0 = No).

*Marijuana use*: if adolescents reported ever having smoked marijuana at least once in their lifetime (1 = used marijuana on at least their lifetime, 0 = did not use marijuana in their lifetime).

*Ever used hard drugs*: if adolescents reported having ever used any one of the following drugs; sniffed glue, mandrax, cocaine, heroin, injection drugs, and any other illegal drug (1 = ever used the drug, 0 = never or did not use the drug) for each of the drugs. Methaqualone is sold under the brand name Quaalude in the US and Mandrax in the UK and South Africa. It was used to reduce anxiety and tension when it was legal as a sedative and hypnotic drug. Due to its addictive nature, it is now illegal for medical use and commonly used by recreational drug users [34]. Mandrax is amongst the frequently abused drugs used by drug users in SA, hence its inclusion in this study.

### Psychological well-being

*Have you had serious sad and hopeless feelings in past 6 months:* if adolescents reported that they felt so sad that they stopped doing their usual activities for 2 weeks or more in a row (1 = Yes, 0 = No).

*Suicidal:* if adolescents reported that they have thought or made a plan about how to attempt suicide during the 6 months preceding the survey (1 = Yes, 0 = No).

## Ethical approval and consent

Ethical Approval for the study was obtained from the South African Medical Association Research Ethics Committee. Active informed consent to conduct the study was obtained from the National Department of Education, school principals, parents and learners. In addition, assent was also obtained from learners on the day of the study. Learners were requested not to write their names on the answer sheet to ensure their anonymity. To ensure learners’ confidentiality, only trained survey administrators were allowed to remain with the learners while completing the survey. School teachers were requested to leave the classroom during data collection time. Learners were also requested not communicate with each other or look at the answer sheet of their peers during the completion of the survey. Information sheet regarding the surveys was also provided to the learners.

## Analyses

Frequency data were weighted to approximate province level distributions of gender and grade, and to account for non-response and province size. Weights were post-stratified by grade and gender, so that the weighted counts of students in each grade and gender combination were in proportion to the provincial population proportions. Each province was to be represented equally in the sample. To account for the variations in provinces’ total population, respondents in highly populated provinces had to have higher weights than respondents from less populated provinces. The three different YRBS data sets were combined for the trend analysis purposes. The trend analysis followed the protocol on Conducting Trend Analysis of YRBS data published by the US Center for Disease Control [35]. Descriptive statistics were first explored to gain a clearer picture of the data as well as to summarize the characteristics of the overall sample. The combined dataset was then split according to the respective years. Frequencies were run for each survey to provide overview differences in characteristics for the three surveys. This provided insight into trends in teenage pregnancy and sexual activity behaviours among adolescent who reported to have ever had sex in the three different surveys. Trends were considered significant if 95 % confidence intervals did not include 1 and the p-value was below 0.05. Bivariate correlation analysis was performed to establish risky behaviours associated with teenage pregnancy.

A binary logistic regression analysis was used to examine unique associations between the demographic, substance use, violence, and sexual and suicide related behaviour variables, and the primary outcome measure: *ever been pregnant.*

Collinearity statistics showed the variables taken into the logistic regression model did not violate the multi-collinearity assumptions: all the variables in the model were below the cut off value for the variance inflation factor (VIF) of 10 [36, 37]. The adjusted relationships of these measures for ever been pregnant were modelled in a binary logistic regression model. A significance level of 5 % was used for all analyses. IBM SPSS Version 21.0 was used to analyse the data. Description of sexual risk behaviours, bivariate correlations, and the logistic regression analysis (Tables 2, 3 and 4) only included adolescent girls (*N* = 5060) who reported to have ever had sex.

## Results

### Demographics

A total of 31 816 school-going adolescents were surveyed during the three surveys (2002, 2008, and 2011). The combined sample consisted of 47.6 % males and 52.4 % females between the ages of 11 to 19 years of age, with (78.4 %) being Black Africans, (13.0 %) Coloured, (1.3 %) Indian, (6.3 %) White, and (1.0 %) other. Less than half of the adolescents have a father (39.0 %) or a mother (31.3 %) who had a paid job.

### Sexual behaviours among adolescents

There has been a decrease in sexual intercourse between the adolescents surveyed in 2002, 2008, and those surveyed in 2011 (41.9 %, 95 % CI: 0.41–0.43; 37.6 %, 95 % CI: 0.36–0.37; and 36.9 %, 95 % CI: 0.35–0.37; respectively) and overall 38.8 %, reported to have ever had sex in the combined sample.

Table 1 shows descriptive characteristics of boys and girls, by year and for the combined sample. There are notable differences on ever had sex between boys and girls throughout the survey years. About 51 % of boys in 2002 reported to ever had sex compared to 34.0 % of girls in the same survey year. Sex among boys and girls in the study continued to decrease from 2002 50.9 %, (95 % CI: 0.48–0.50), 2008 45.1 %, (95 % CI: 0.53–0.56), to 2011 45.2 %, (95 % CI: 0.53–.0.56) for boys; and 34.0 %, (95 % CI: 0.65–0. 67); 30.1 %, (95 % CI: 0.69–0.71); 29.8 %, (95 % CI: 0.69–0.71) for girls in 2002, 2008, and 2011, respectively. In the combined sample similar proportions of both boys (11.1 %), and girls (10.9 %) reported to ever making someone pregnant for boys or ever having been pregnant in the case of girls. Overall, 11.0 % of the adolescents reported to ever made someone pregnant or ever been pregnant in this study.

**Table 1:** Descriptive characteristics of the total sample^a^ by year

BOYS					GIRLS				Total sample			
Characteristics	2002 (*N* = 4930) %	2008 (*N* = 5106) %	2011 (*N* = 5166) %	TOTAL BOYS (*N* = 15202) %	2002 (*N* = 5619) %	2008 (*N* = 5164) %	2011 (*N* = 5831) %	TOTAL GIRLS (*N* = 16614) %	2002 (*N* = 10549) %	2008 (*N* = 10270) %	2011 (*N* = 10997) %	ALL (*N* = 31 816) %
Age									-	-	-	-
Mean	16.9	16.1	16.6	16.6	16.4	16.1	16.3	16.2	16.6	16.1	16.4	16.4
Standard deviation	3.3	1.9	2.0	2.5	2.6	1.9	1.9	2.2	2.9	1.9	1.9	2.3
Gender												
Male	-	-	-	-	-	-	-	-	46.7	49.1	47.0	47.6
Female	-	-	-	-	-	-	-	-	53.3	50.9	53.0	52.4
Race												
Black African	74.7	78.7	82.4	78.7	73.5	79.1	82.3	78.3	74.1	78.5	82.4	78.4
Coloured	14.4	13.7	10.1	12.6	15.6	14.8	10.0	13.4	15.0	14.2	10.0	13.0
Indian	1.4	0.9	1.4	1.2	1.2	0.8	1.3	1.1	1.3	1.2	1.4	1.3
White	8.4	6.0	4.7	6.4	8.8	4.4	5.3	6.2	8.7	5.3	5.1	6.3
Other	1.1	0.7	1.4	1.1	0.9	0.9	1.1	0.9	0.9	0.8	1.2	1.0
Socio-economic status												
Father has paid job 5 or more days a week	41.1	40.5	37.9	39.8	40.2	38.1	36.4	38.2	40.6	39.4	37.1	39.0
Yes, works less than 5 days a week	8.6	9.2	8.5	8.7	8.5	7.4	7.4	7.7	8.5	8.3	7.9	8.2
No, he gets a social grant	^b^	7.0	7.3	10.6	^b^	7.3	7.3	10.7	^b^	7.1	7.3	10.6
No, he is unemployed	17.5	10.9	11.1	8.2	17.4	12.2	12.0	8.9	17.5	11.6	11.6	8.6
No, he is ill or disabled	2.6	1.4	1.1	2.7	2.7	1.6	0.9	2.2	2.6	1.5	1.0	2.4
No, he has retired	5.5	3.5	3.0	7.6	4.1	2.7	2.3	7.9	4.8	3.1	2.6	7.7
My father is deceased	16.5	10.0	13.3	10.6	18.4	13.0	14.7	12.1	17.5	11.5	14.1	11.4
I don’t know	8.2	17.4	17.8	11.8	8.7	17.8	19.0	12.1	8.5	17.6	18.4	12.0
Mother has paid job 5 or more days	29.8	32.4	31.2	31.2	32.4	30.8	31.1	31.4	31.2	31.7	31.1	31.3
Yes, works less than 5 days a week	12.6	12.7	12.3	12.5	11.1	12.1	10.8	11.3	11.8	12.4	11.5	11.9
No, she gets a social grant	23.3	9.5	10.7	14.4	24.8	11.6	12.1	16.3	24.1	10.5	11.4	15.4
No, she is a housewife	20.0	21.9	20.5	20.8	19.6	24.1	23.8	22.5	19.9	22.9	22.2	21.7
No, she is ill or disabled	2.1	2.0	1.7	2.0	1.9	1.8	1.7	1.8	2.0	1.9	1.7	1.9
No, she has retired	3.6	3.8	3.7	3.7	2.3	2.5	3.1	2.6	2.9	3.1	3.4	3.1
My mother is deceased	4.8	5.5	7.4	5.9	5.1	6.5	7.8	6.5	5.0	6.0	7.6	6.2
I don’t know	3.6	12.2	12.5	9.5	2.8	10.6	9.7	7.6	3.2	11.4	11.0	8.5
Sexual behaviours												
Ever had sex	50.9	45.1	45.2	47.0	34.0	30.1	29.8	31.3	41.9	37.6	36.9	38.8
^a^Ever been pregnant	11.0	10.7	11.6	11.1	11.2	11.2	10.3	10.9	11.1	11.0	10.9	11.0
Violent behaviours												
Ever been hit by boyfriend or girlfriend	15.3	24.7	19.2	19.2	13.6	21.1	17.2	16.6	14.4	23.0	18.2	17.9
Ever hit your boyfriend or girlfriend	15.6	21.8	18.1	18.2	11.4	17.6	13.9	13.8	13.4	19.7	15.9	15.9
Ever been forced to have sex	8.7	10.9	10.7	10.1	10.7	8.7	8.3	9.2	9.8	9.9	9.4	9.7
Ever forced someone to have sex	9.7	11.6	11.3	10.9	6.3	6.4	5.1	5.9	7.9	9.0	8.0	8.3
Cigarette smoking												
0 days	67.1	71.5	75.4	71.4	79.9	83.1	86.1	83.1	73.9	77.2	81.1	77.5
1 or more days	32.9	28.5	24.6	28.6	20.1	16.9	13.9	16.9	26.1	22.8	18.9	22.5
Alcohol use												
1 or more days	58.9	58.4	57.6	58.3	49.1	47.3	50.4	49.0	53.7	52.6	46.2	50.8
Binge drinking												
1 or more day	31.7	36.8	33.7	34.1	21.1	24.9	24.0	23.3	26.1	30.8	28.6	28.5
Drugs												
Marijuana	23.4	19.8	19.6	20.9	9.6	8.8	7.6	8.7	16.1	14.4	13.2	14.6
Sniffed glue	11.7	14.7	13.9	13.5	7.5	7.5	6.7	7.2	9.5	11.4	10.1	10.3
Mandrax	7.7	9.4	8.1	8.4	4.3	6.1	3.2	4.5	5.9	8.2	5.5	6.5
Cocaine	7.3	8.8	7.7	7.9	3.8	4.1	3.2	3.7	5.4	6.9	5.3	5.9
Heroin	11.1	7.6	8.5	9.1	9.1	4.4	2.9	5.4	10.0	6.4	5.5	7.3
Injection	7.6	9.2	8.9	8.6	4.9	5.1	3.7	4.5	6.2	7.5	6.1	6.6
Other	7.5	9.3	5.8	7.5	4.6	4.5	2.4	3.8	6.0	7.3	4.0	5.7
Psychological well-being												
Ever had serious sad and hopeless feelings	24.8	24.5	24.3	24.5	25.1	24.0	25.7	24.9	25.0	24.4	25.0	24.8
Suicidal	17.3	18.4	16.3	17.3	21.8	23.2	21.0	22.0	19.7	21.1	18.8	19.9

^a^Where boys were asked whether they have made someone pregnant and girls were asked if they ever been pregnant before

^b^ “No, he gets a social grant” was not available as an option in 2002 survey

### Violent behaviours among adolescents

About 17.9 % of the adolescents reported having been hit on purpose by a boyfriend or girlfriend, while 15.9 % reported to ever hit their boyfriend or girlfriend on purpose. During the three survey years, 9.8 %, 9.9 %, and 9.4 % of the adolescents reported that they had been forced to have sex, respectively. In the combined sample, 9.7 % of adolescents reported having been forced to have sex, while 8.3 % reported to have forced someone else to have sex.

### Substance use related behaviours among adolescents

Ever smoked cigarettes was reported by 22.5 % of the adolescents, ever having used alcohol by 53.4 % and 28.5 % admitted to binge drinking. Smoking dagga (marijuana) was reported by (14.6 %) adolescents, followed by sniffed glue (10.3 %), heroin (7.3 %), and injection drugs (6.6 %). Mandrax, cocaine and other drugs were the least used drugs by the adolescents at 6.5 %, 5.9 % and 5.7 % respectively.

When comparing the different years, marijuana use decreased from 16.1 % (95 % CI: 0.15–0.17) in 2002, 14.4 % (95 % CI: 0.14–0.15) in 2008 to 13.2 % (95 % CI: 0.13–0.14) in 2011. The use of mandrax increased in 2008 to 8.2 % (95 % CI: 0.08–0.09) from 5.9 % (95 % CI: 0.05–0.06) in 2002, although it decreased in 2011 to 5.5 % (95 % CI: 0.05–0.06). This pattern was also observed for the use of cocaine in 2002, 2008 and 2011 at 5.4 % (95 % CI: 0.05–0.06), 6.9 % (95 % CI: 0.06–0.07) and 5.3 % (95 % CI: 0.05–0.06); respectively.

Substance use between boys and girls were notably different. Nearly twenty nine per cent of boys (28.6 %) smoked cigarettes one or more days, while only 16.9 % of girls smoked cigarettes. More boys (58.3 %) than girls (49.0 %) ever used alcohol in this study. Similarly with binge drinking during the past month, 34.1 % boys and 23.3 % girls reported binge drinking. When comparing the three surveys, there is a slight decrease in alcohol use from 2002 (53.7 %), 2008 (52.6 %) to 2011 (46.2 %). The overall use of alcohol in the combined sample is 50.8 %. The percentage of binge drinkers was 26.1 % in 2002, but increased in 2008 (30.8 %) and slightly decreased in 2011 (28.6 %). The overall percentage of binge drinking in the combined sample was 28.5 %. Marijuana, the drug most commonly used by the adolescents in this study was reported by 20.9 % of the boys and by 8.7 % of the girls. Sniffed glue (13.5 %), mandrax (8.4 %), cocaine (7.9 %), heroin (9.1 %), injection drugs (8.6 %), and other illegal drugs (7.5 %) were reported by the boys. While girls reported to have ever sniffed glue (7.2 %), used mandrax (4.5 %), cocaine (3.7 %), heroin (5.4 %), injection drugs (4.5 %), and other illegal drugs (3.8 %).

### Sexual risk behaviours and teenage pregnancy

Table 2 below shows trends in sexual risk behaviours and teenage pregnancy among girls who reported to have ever had sex. The prevalence of ever been pregnant among the adolescent girls who ever had sex had increased from 17.3 %, (95 % CI: 0.16–0.19) in the 2002 survey, 23.6 %, (95 % CI: 0.21–0.26) in 2008, and slightly decreased in 2011 to 21.3 %, (95 % CI: 0.19–0.23). This shows a significant (*p* < 0.01) increasing trend from the 2002 survey to the 2008 survey. The overall prevalence rate of ever been pregnant among the adolescent girls who reported to ever had sex was 20.5 %. Among those who reported ever had sex, 8.6 % in 2002, 6.6 % in 2008 and 7.4 % in 2011 had sex at the age of 13 years or younger, and with the overall being 7.7 % when combining the three surveys. Sex with one person was reported by 68.2 % of the girls, while 31.8 % reported having had sex with 2 or more persons. When comparing the three surveys, 30.2 % of the girls in 2002, 28.7 % in 2008, and 34.9 % in 2011 reported to have sex with 2 or more persons.

**Table 2:** Sexual risk behaviours among adolescent girls who reported to ever had sex in their lives

Risky behaviours	2002	2008	2011	ALL
	(*N* = 1854)	(*N* = 1499)	(*N* = 1707)	(*N* = 5060)
	%	%	%	%
First had sex at age ≤ 13	8.6	6.6	7.4	7.7
Number of sexual partners				
1 partner	68.8	69.9	62.7	68.2
2 or more partners	30.2	28.7	34.9	31.8
Ever use alcohol before sex	6.7	13.1	13.8	11.0
used condom during sex				
Never or rarely use condoms	32.8	25.4	27.6	28.9
Mostly use condoms	67.2	74.6	72.4	71.1
Contraceptive methods				
No method	16.0	14.2	13.3	14.6
Birth control pills	4.6	3.8	5.2	4.6
Condoms	32.5	44.3	46.6	40.7
Injection	14.4	11.7	12.6	13.0
Other	8.2	7.5	8.7	8.1
Ever been pregnant	17.3	23.6	21.3	20.5
Ever had an abortion	5.1	6.8	6.0	5.9

Having sex under the influence of alcohol was reported by 6.7 % of the girls in 2002, 13.1 % in 2008, and 13.8 % in 2011. The overall rate was 11.0 % when all three surveys combined. This shows an increasing pattern of sexual intercourse under the influence of alcohol, as well as the use of alcohol. The prevalence of abortion was reported by 5.1 % in 2002, 6.8 % in 2008, and 6.0 % in 2011. The overall rate of condom use during sex was reported by 71.1 % of the girls. Furthermore, condom use increased from 67.2 % (95 % CI: 0.79–0.81) in 2002 to 74.6 %, (95 % CI: 0.84–0.85) in 2008, but decreased slightly in 2011 to 72.4 % (95 % CI: 0.84–0.85). With regard to contraceptive methods used, condoms were among the most frequently used method compared to any other methods used at 40.7 %, while birth control pills were among the least used methods at 4.6 %. When comparing the surveys, 32.5 % of the girls in 2002, 44.3 % in 2008, and 46.6 % in 2011 reported the use of condoms as the method for contraception. Use of birth control pills, on the one hand were reported by 4.6 %, 3.8 %, and 5.2 % in 2002, 2008, and 2011; respectively. The overall use of birth control pills in the combined sample was 4.6 %. On the other hand, injection use was reported by 14.4 %, 11.7 %, and 12.6 % in 2002, 2008, and 2011; respectively. The overall use of injection was 13.0 % in the combined sample. In the combined sample, 14.6 % reported no method used for contraceptive purposes.

### Association between teenage pregnancy and sexual risk behaviours

The associations between the teenage pregnancy and risky behaviours were examined for those reported to have ever had sex. Table 3 below shows the results of the Pearson correlations between teenage pregnancy and the risky behaviours. There was a negative association between teenage pregnancy and condom use during sex (*r* = -.084, *p* < 0.01). This negative association, although weak, shows that when teenagers practise protective behaviours such as using condoms during sex; they are less likely to become pregnant.

**Table 3:** Association between teenage pregnancy and sexual risk behaviours among adolescent girls who ever had sex (*N* = 5060)

Risky behaviours	Ever been pregnant (r)
First had sex at age ≤13	.056^b^ (95 % CI: 0.03–0.08)
Number of sexual partners	.033^a^ (95 % CI: 0.01–0.04)
Ever used alcohol before sex	.012 (95 % CI: 0.02–0.04)
Always used a condom during sex	-.084^b^ (95 % CI: -0.11–-0.06)
Ever been forced to have sex	.032^a^ (95 % CI: 0.01–0.06)
Ever forced someone to have sex	.046^b^ (95 % CI: 0.02–0.07)
Ever been hit by boyfriend	.048^b^ (95 % CI: 0.01–0.08)
Ever hit your boyfriend	.057^b^ (95 % CI: 0.03–0.08)

^a^ Correlation is significant at the 0.05 level (2-tailed)

^b^ Correlation is significant at the 0.01 level (2-tailed)

The age at first sex, which is the age of 13 years or younger (*r* = .056, *p* < 0.01), and the number of sexual partners being two or more sexual partners (*r* = .033, *p* < 0.05), were positively correlated with teenage pregnancy. Ever been forced to have sex (*r* = .032, *p* < 0.05), as well as ever forced someone to have sex (*r* = .046, *p* < 0.01), and been hit by boyfriend (*r* = .048, *p* < 0.01) were also positively correlated with teenage pregnancy.

### Risk behaviours predictive of teenage pregnancy

Logistic regression analysis (Table 4) showed significant associations between race and teenage pregnancy with being White having lower odds of ever been pregnant (OR: 0.374, 95 % CI: 0.203–0.691) than being Black African. Girls who engaged in first sexual intercourse at the age 13 years or younger had higher odds (OR: 1.299; 95 % CI: 0.812–2.078) of ever been pregnant than the girls who first had sex at the age 14 years or older. Having sex with 2 or more sexual partners had higher odds (OR: 1.250; 95 % CI: 1.039–1.503) of ever been pregnant than girls who had just one sexual partner. Condom use, as a contraceptive method used to prevent pregnancy had lower odds (OR: 0.462; 95 % CI: 0.309–0.691) of ever been pregnant compared to other methods of contraceptives used by the girls in this study. Girls using injection (OR: 1.142, 95 % CI: 0.747–1.746) or birth control pills (OR: 1.004, 95 % CI: 0.618–1.762) for contraception had higher odds of ever been pregnant.

**Table 4:** Results of binary logistic regression analyses on ever been pregnant (dependent variable) with socio-demographics and risk behaviours (independent variables) from adolescent girls who ever had sex (*N* = 5060)

	Have you ever been pregnant		
Variables	N	OR	95 % CI
Demographic characteristics			
Race			
Black African (Reference)	3261		
Coloured	408	1.047	0.762–1.439
Indian	30	0.649	0.219–1.917
White*	**201**	**0.374**	**0.203–0.691**
Other	30	1.676	0.738–3.810
Age (in years)			
≤ 13 (Reference)	58		
14	130	0.891	0.416–1.909
15	258	0.748	0.371–1.507
16	352	0.675	0.340–1.341
17	361	0.619	0.311–1.232
≥ 18	2771	0.724	0.384–1.367
Socio-economic status			
Does your father have a paying job			
Yes, works 5 or more days a week (Reference)	1383		
Yes, works less than 5 days a week	292	0.907	0.641–1.283
No, he gets a social grant	479	1.019	0.768–1.352
No, he is unemployed*	**354**	**1.430**	**1.061–1.928**
No, he is ill or disabled	113	1.483	0.922–2.384
No, he has retired	423	0.807	0.594–1.097
My father is deceased*	**498**	**1.451**	**1.112–1.893**
I don’t know	388	1.119	0.814–1.537
Does your mom have a paying job			
Yes, works 5 or more days a week (Reference)	1200		
Yes, works less than 5 days a week	443	1.007	0.747–1.358
No, she gets a social grant	690	1.195	0.929–1.537
No, she is a housewife	914	0.962	0.757–1.222
No, she is ill or disabled	82	1.132	0.641–1.999
No, she has retired	90	1.218	0.711–2.089
My mother is deceased	289	1.134	0.811–1.586
I don’t know*	**222**	**1.484**	**1.018–2.163**
Sexual activity and related behaviours			
First sex at age ≤ 13	267	1.299	0.812–2.078
Number of sexual partners			
1 person (Reference)	2599		
2 or more persons*	**1331**	**1.250**	**1.039–1.503**
Ever use alcohol before sex*	**407**	**1.373**	**1.004–1.878**
Condom use			
Rarely (Reference)	1195		
Mostly*	**2735**	**0.790**	**0.649–0.962**
Contraceptive methods			
No method (Reference)	570		
Birth control pills	160	1.004	0.618–1.762
Condoms*	**1697**	**0.462**	**0.309–0.691**
Injection	557	1.142	0.747–1.746
Other*	**341**	**0.602**	**0.370–0.978**
Violence			
Ever been hit by your boyfriend	950	1.204	0.975–1.488
Ever hit your boyfriend	693	1.247	0.987–1.575
Ever forced to have sex	655	0.974	0.768–1.235
Ever forced someone to have sex	287	1.165	0.845–1.607
Psychological well-being			
Ever had serious sad and hopeless feelings	1349	1.075	0.898–1.287
Suicidal	1086	0.938	0.808–1.196
Smoking and substance use related behaviours			
Smoked cigarettes*	**869**	**0.641**	**0.497–0.825**
Alcohol use	2451	0.871	0.718–1.057
Binge drinking*	**1331**	**0.624**	**0.503–0.774**
Marijuana	466	1.315	0.970–1.782
Sniffed glue,	329	0.979	0.711–1.348
Mandrax*	**135**	**1.968**	**1.243–3.117**
Cocaine	123	1.549	0.935–2.566
Heroin	211	1.021	0.696–1.499
Injection	154	1.176	0.764–1.808
Other	150	0.839	0.510–1.380

*Bold means the variable is significant, *p* < 0.05

Among the smoking and substance use related behaviours; smoking cigarettes (OR: 0.641, 95 % CI: 0.497–0.825), binge drinking (OR: 0.624, 95 % CI: 0.503–0.774) and mandrax use (OR: 1.968, 95 % CI: 1.243–3.117) had higher odds as predictors of ever been pregnant during teenage years. Thus, adolescents smoking cigarettes and or using these drugs were more likely to ever been pregnant, than adolescent who were non-users.

## Discussion

The first important finding in this study is that sexual intercourse among adolescents has decreased. However, among those adolescent girls who reported to engage in sex, teenage pregnancy has increased. These findings are in line with increasing evidence demonstrating the magnitude of teenage pregnancy among sexually active adolescents, especially in the sub-Saharan African countries [1, 2, 4, 19, 20]. This is worrying, particularly the fact that it suggest that there is little progress made in reducing teenage pregnancy over the past few decades, despite its importance as highlighted in the Millennium Development Goals (MDG) and its association to maternal mortality and morbidity rates [5]. Teenage pregnancy in developing countries has been reported to be worse than in developed countries [4, 5]. The lack of data on teenage pregnancy trends among South African adolescents poses a serious public health threat, as the magnitude of the problem is relatively hidden for consideration into intervention programs that aim to reduce teenage pregnancy, and improve maternal and child health outcomes. The fluctuating rates of sexual intercourse at the age of 13 years or younger among girls is also concerning. Early sexual debut is not uncommon in SA. Recent research shows that more and more adolescents are sexually active by the time they are 14 years and older [25, 38].

This study shows that the trends in teenage pregnancy have remained steady from 2002 to 2011. When looking at those girls who reported to ever had sex, teenage pregnancy has even increased from 2002 (17.3 %) to 2011 (21.3 %). The increased pregnancy rates among these girls can be attributed to, among other factors, the fewer girls who are using contraceptive methods to prevent pregnancy but are sexually active in this study, despite the fact that contraceptives are available for free at the public healthcare settings in the country. Contraceptives, which are directly provided from the healthcare setting, such as birth control pills and contraceptive injections, are among the least of contraceptive methods used by adolescent girls in this study. The findings of small proportion of girls using modern contraceptives, such as birth control pills is surprising given the fact that contraceptives are provided free of charge in SA [13–15]. However, this can be explained by (1) the poor knowledge of contraceptives among adolescents in general in SA [29, 38], and (2) the limited availability of, and access to sexual and reproductive health services targeted at adolescents [39, 40]. Furthermore, the cultural or societal, structural, and individual factors associated with the use of contraceptives are some of the barriers for contraceptive use [40, 41]. These include, the need to prove fertility and maturity by the girls, opposition from male partners, perception of personal low risk around the ability to fall pregnant, and fear of side effects [12, 16, 41–43]. On one hand, low levels of contraceptive use and the attitude toward using them among adolescents has been associated with high rates of unwanted pregnancies and unsafe abortions among youth in Sub-Saharan Africa [40, 42]. On the other hand, low levels of contraceptive use has also been attributed to limited capacity of the health care system, particularly in rural settings and structures within which family planning services are offered [6]. There is increasing evidence on the youth unfriendliness of the primary health care facilities in the country, which in turn might be a contributing factor to the poor use of contraceptives among adolescents; and thus exacerbate the trends in teenage pregnancy in SA [43–45].

The second important finding in this study is that girls who use condoms when having sexual intercourse were less likely to get pregnant as a teenager. This finding supports previous research on reported knowledge and potential benefits of consistent and correct condom use by most young people in SA [43, 44]. It is well known that condom use during sexual intercourse not only prevents unwanted pregnancy; but also prevents contraction of HIV and other STIs [43, 44]. This finding also shows that the efforts made by the government in making condoms free of charge and easily accessible are beneficial to the youth of SA [9, 43, 45]. This is also in line with research findings from HICs where decline in teenage pregnancy is attributed to improved contraceptive use, including condom use [46]. Therefore, continuous efforts on encouraging and facilitating condom use among young people in SA still proves to be an important behavioural intervention worth investing in, both for the prevention of HIV and other STIs’ transmission and for contraceptive purposes.

Thirdly, girls who have sex with only one sexual partner in this study were also less likely to fall pregnant compared to the girls who had 2 or more sexual partners during their teenage years. Multiple sex partners is common in SA and has gender and cultural connotations, especially in KwaZulu Natal (KZN) and the Eastern Cape (EC) rural communities [9, 44, 45, 47]. Having more than one sexual partner for boys in KZN, as well as in the EC proves “masculinity” among boys, while for some girls it guarantees peer approval although it has to be discreet among girls [6, 9, 44, 47, 48]. This highlights another important area needing intervention for behaviour change, since a quarter of the girls in this study reported to have sex with 2 or more sexual partners.

The fourth and last finding of this study is the larger proportion of adolescents using alcohol and drugs. Clearly, alcohol and substance use among adolescents also requires attention. About half of the adolescents in this study have used alcohol one or more times in their lives. Substance use, in a few studies has been linked to teenage pregnancy. A study in Sweden reported that smoking and binge drinking among males was associated with the likelihood of the males having made their partners pregnant [49]. In this study, girls who never use alcohol before having sex, not practice binge drinking, who do not smoke cigarettes and do not use mandrax were less likely to fall pregnant as a teenager. The risk factors associated with teenage pregnancy, including alcohol use, smoking (tobacco or drug use) were also reported by Imamura et al., (2007) [50]. The author reported that these health risk behaviours tend to co-occur with teenage pregnancy in the European countries. Some studies have also reported on the possible link between teenage pregnancy and alcohol, and or tobacco and drug use [50–52]. The findings of this particular study show that substance use increases the likelihood of adolescents falling pregnant during their teenage years. This is in line with previous findings that these risk behaviours, especially alcohol use, have been associated with other sexual-risk taking behaviours that result in pregnancy [53]. This study also found that girls who had a father who is unemployed or deceased were more like to get pregnant; and in terms of race, girls who belonged to the White race were less likely to become pregnant than other races. This finding supports previous research where low socio-economic status was found to be associated with teenage pregnancy in SA [20, 26, 27]. It is well documented in SA that teenage pregnancy is much more prevalent among the poor Black Africans [20, 26].

A limitation of the study at hand is that it is undertaken in the school setting. This excludes those adolescents who were not schooling at the time of data collection. Teenage pregnancy has been reported to be the most common reason for school-dropout in SA [26, 28]. Therefore, some adolescents might be out of school as a result of pregnancy and that does not invalidate the findings of this study, which confirms teenage pregnancy is still prevalent among adolescents in SA. Another limitation is that the results of this study cannot prove causality of teenage pregnancy by these risk behaviours, but only associations with teenage pregnancy. Thus, future research should aim to mitigate the risk behaviours associated with teenage pregnancy, as reported in this study. The pattern in teenage pregnancy is clearly increasing among those who engage in sexual activities, and that is a cause for concern. This suggests the further need to address health risk behaviours, including early sexual debut, unsafe sex, substance use and encouraging the correct and consistent use of contraceptives among adolescents; as well as promote their sexual and reproductive health in SA.

## Conclusions

Sexual intercourse among adolescents is decreasing, but teenage pregnancy continues to increase among those who are sexually active. Teenage pregnancy continues to be a growing public health concern and adolescent sexual and reproductive health behaviours, such as contraceptives and safe sex practices has to be acknowledged in the health care system as an important health issue in SA. Risky behaviours, such as substance use, also need to be recognized and intervention programmes aiming to reduce teenage pregnancy and sexual and reproductive behaviours among adolescents need to include prevention of substance use. This study highlights the need for more comprehensive research on adolescents SRH needs particularly among the vulnerable adolescents aged 16 years and under, but not excluding those aged above 16 years old, to identify risk factors and develop specific interventions tailored for their needs. Regular monitoring of teenage pregnancy trends and the associated factors also aid in determining the effectiveness of programmes put in place by government and other related institutions, as well as highlighting areas where extra effort is needed to curb the rates of teenage pregnancy.
